# Outcome reporting in randomized controlled trials (RCTs) on the pharmacological management of idiopathic overactive bladder (OAB) in women; a systematic review for the development of core outcome sets (COS)

**DOI:** 10.1007/s00192-021-05040-1

**Published:** 2022-01-10

**Authors:** Reem Moussa, Maria Patricia Rada, Constantin Durnea, Gabriele Falconi, Cornelia Betschart, Jorge Milhem Haddad, Philip Sedgwick, Stergios K. Doumouchtsis, CHORUS: An International Collaboration for Harmonising Outcomes, Research and Standards in Urogynaecology and Women’s Health (i-chorus.org)

**Affiliations:** 1 Institute of Medical and Biomedical Education, St George’s University of London, London, UK; 2 2nd Department of Obstetrics-Gynaecology, “luliu Hatieganu”, University of Medicine and Pharmacy, Cluj-Napoca, Romania; 3 Department of Obstetrics and Gynaecology, Luton and Dunstable University Hospital NHS Foundation Trust, Luton, UK; 4 Complex Operative Unit of Gynecology, Fondazione Policlinico Tor Vergata University Hospital, Rome, Italy; 5 University Hospital and University of Zurich, Zurich, Switzerland; 6 Hospital das Clinicas da Faculdade de Medicina da, Universidade de São Paulo, São Paulo, Brazil; 7 Department of Obstetrics and Gynaecology, Epsom & St Helier University Hospitals NHS Trust, London, UK; 8 Laboratory of Experimental Surgery and Surgical Research N S Christeas, National and Kapodistrian University of Athens, Medical School, Athens, Greece; 9 American University of the Caribbean, School of Medicine, Pembroke Pines, Florida, USA; 10 Ross University, School of Medicine, Miramar, Florida, USA

**Keywords:** Overactive bladder; core outcome set, Urgency urinary incontinence, Urinary incontinence, Urinary urgency, Pelvic floor disorders, Outcomes, LUTS

## Abstract

**Introduction and hypothesis:**

Evidence on OAB management remains suboptimal and methodological limitations in randomized control trials (RCTs) affect their comparability. High quality meta-analyses are lacking. This study aimed to compare selection and reporting of outcomes and outcome measures across RCTs as well as evaluate methodological quality and outcome reporting quality as a first stage in the process of developing core outcome sets (COS).

**Methods:**

RCTs were searched using Pubmed, EMBASE, Medline, Cochrane, ICTRP and Clinicaltrials.gov from inception to January 2020, in English language, on adult women. Pharmacological management, interventions, sample size, journal type and commercial funding were documented. Methodological and outcome reporting quality were evaluated using JADAD and MOMENT scores.

**Results:**

Thirty-eight trials (18,316 women) were included. Sixty-nine outcomes were reported, using 62 outcome measures. The most commonly reported outcome domains were efficacy (86.8%), safety (73.7%) and QoL (60.5%). The most commonly reported outcomes in each domain were urgency urinary incontinence episodes (UUI) (52.6%), antimuscarinic side effects (76.3%) and change in validated questionnaire scores (36.8%).

A statistically significant correlation was found between JADAD and MOMENT (Spearman’s rho = 0.548, *p* < 0.05) scores. This indicates that higher methodological quality is associated with higher outcome reporting quality.

**Conclusions:**

Development of COS and core outcome measure sets will address variations and lead to higher quality evidence. We recommend the most commonly reported outcomes in each domain, as interim COS. For efficacy we recommend: UUI episodes, urgency and nocturia episodes; for safety: antimuscarinic adverse events, other adverse events and discontinuation rates; for QoL: OAB-q, PPBC and IIQ scores.

**Supplementary Information:**

The online version contains supplementary material available at 10.1007/s00192-021-05040-1.

## Introduction

Overactive bladder (OAB) is a common condition that carries a significant impact on women’s quality of life (QoL) along with a high economic burden [1]. Medical therapy offered can vary extensively and there is a lack of sufficient data to prove the efficacy of each drug prescribed. In reviews that assessed pharmacological therapy, there was no statistically significant difference between the efficacy of drugs trialled [2]. Additionally, Cochrane reviews raised concerns about standardization of QoL outcomes, patient reported outcomes (PROs), economic outcomes and overall outcome reporting and outcome measures [3] . Unless a patient-centred approach is established in clinical research, reduced patient satisfaction despite improvement in symptoms may complicate the development of clinical pathways. The lack of consistency in outcome reporting for OAB and other gynaecological conditions can produce data that are less comparable and robust and can slow the progression within this field by preventing the effective synthesis of data for high quality meta-analyses [4]. In order to support higher quality research evidence, the development of ‘core outcome sets’ (COS) and ‘core outcome measures sets (COMS) is essential. These efforts have been supported by the Core Outcome Measures in Effectiveness Trials (COMET) initiative and COS have been developed in many areas of research as well as clinical practice [5].

The COS-STAD Statement proposes that systematic reviews should be undertaken to identify outcomes to be included in a subsequent consensus process [6].

The aim of this systematic review was to evaluate variation of outcome and outcome measure reporting in randomized controlled trials (RCT) on the pharmacological management of idiopathic OAB in women, in order to develop an inventory of potentially eligible core outcome and outcome measure sets as a first stage of this process.

In addition to identifying such outcomes, and creating an inventory to inform the process described above, we aimed to evaluate the outcome reporting quality and methodological quality of the included trials. Methodological parameters were assessed against publication characteristics.

## Methods

This study was undertaken by one of the Working Groups of CHORUS, An International Collaboration for Harmonising Outcomes, Research and Standards in Urogynaecology and Women’s Health (i-chorus.org). It is part of wider projects led by CHORUS, applying information and data obtained from primary research for the development of Core Outcome Sets (COS) and Core Outcome Measures Sets (COMS) to be used in future research into pelvic floor disorders [7–20]. This project has been registered with COMET Initiative (Reg. No 981).

This study was undertaken by one of the Working Groups of CHORUS, An International Collaboration for Harmonising Outcomes, Research and Standards in Urogynaecology and Women’s Health (i-chorus.org). It is part of wider projects led by CHORUS, applying information and data obtained from primary research for the development of Core Outcome Sets (COS) and Core Outcome Measures Sets (COMS) to be used in future research into pelvic floor disorders [7–20]. This project has been registered with COMET Initiative (Reg. No 981).

As this systematic review is part of the process of development of COS and COMS in the field of idiopathic OAB in women, we followed the recommendations and methodology of the COS-STAD Statement as well as established methodology applied and developed in our recent systematic reviews in other areas of pelvic floor disorders [7–20].

### Search strategy

A comprehensive literature search was performed, using MEDLINE and EMBASE, as well as Cochrane Central Register of Controlled Trials (CENTRAL), ICTRP and Clinicaltrials.gov. Inclusion criteria included the following Medical Subject Heading (MeSH) terms: ‘overactive bladder’, ‘idiopathic’, ‘randomised controlled trial’, ‘randomized controlled trial’, ‘urgency’, ‘urge incontinence’, ‘urgency incontinence’, ‘anticholinergic’, ‘antimuscarinic’, ‘mirabegron’, ‘solifenacin’, ‘tolterodine’, ‘oxybutynin’, ‘trospium’ and ‘female’. The search was refined to include articles from the inception to January 2020, written in the English language and carried out only on humans and on adult women. Duplicates were removed. The initial search yielded 1402 results. As well as identifying eligible trials, the Cochrane Central Register was used to identify any Systematic Reviews(SRs) relevant to pharmacological management of OAB, to aid with snowballing.

Snowballing is a methodological technique used to identify additional studies that may be eligible based on the inclusion criteria. This can be achieved by searching the reference list of SRs as well as primary studies.

### Selection criteria

Title screening was followed by abstract screening and full text screening (Supplementary Fig. 1- S.1). We excluded articles that did not meet the inclusion criteria in a standardized and staged approach. The search was narrowed to 530 studies after title screening and subsequently to 38 studies after abstract and full text screening. Any studies that were observational or non-randomized were excluded. Studies that were recruiting, withdrawn or terminated were excluded from the review, as were studies with no results and hence reported outcomes available. Furthermore, any studies that assessed neurogenic OAB or included non-pharmacological interventions (such as bladder retraining) were excluded. Moreover, since only conventional drugs that can be self administered by the woman were of interest, studies focused on phytotherapeutic drugs or drugs (e.g. botulinum toxin type A injections) that cannot be self administered were also excluded. Studies on men were excluded, too. Any article that was not available in full text or not written in the English language was also excluded from the analysis. This process was conducted using the Preferred Reporting Items for Systematic Reviews and Meta-analyses (PRISMA) statement [21].

### Data collection and analysis

Data were extracted from the full text articles, and entered onto a dedicated Excel spreadsheet to develop an inventory. The study size, interventions, primary and secondary outcomes and outcome measures used were all identified and documented. Outcome measures included voiding diaries, laboratory parameters and questionnaires used for QoL assessments. In addition, the journal type, journal name and commercial funding were recorded (Table 3 – S.4). This process was undertaken independently by two researchers (RM and MPR). Outcomes were identified and added to the inventory as verbatim, and subsequently were grouped together into themes, domains and subdomains. This process was conducted in accordance with the COMET Initiative Systematic Reviews Guidance [5], and we followed similar methodology principles previously developed and applied in other CHORUS systematic reviews [7–20].

The full text articles were then independently assessed by two researchers (MPR and CD) using the JADAD criteria and the Management of Otitis Media with Effusion in Cleft Palate (MOMENT) scoring system [22]. The JADAD criteria aim to assess ‘the quality of reporting of RCTs’ [23]. The criteria consist of three questions with a maximum of five points available; these questions relate to the randomisation of the search, blinding and accounting for the fate of all patients in the trial. A modified version of the JADAD scale was developed to include eight questions addressing clearly mentioned adverse effects, a clear description of the inclusion and exclusion criteria and a described method of statistical analysis [24]. The modified JADAD scale was used in this study.

The MOMENT criteria comprise six questions to assess the ‘quality of describing and reporting outcomes’ in RCTs. Questions assess whether primary and secondary outcome(s) have been clearly stated and defined to allow the reproducibility of results, as well as whether an appropriate explanation for the use of outcomes has been given. This facilitates development of a COS to be used in future trials.

### Statistical analysis

The journal type (general, specialist or sub-specialist), the impact factor of the journal of publication in the year of publication was retrieved from InCites (Web of Science, Thomson Reuters) and was documented in the datasheet. These data, as well as the categories of commercial funding (yes or no) were each compared against the JADAD and MOMENT scores using the non-parametric Kruskal-Wallis test. The critical level of significance was 0.05 (5%). Furthermore, the two-tailed Spearman’s rho test was calculated to assess the correlation of MOMENT (quality of outcome reporting), JADAD (quality of methodology), year of publication and impact factor (IF) of the papers. The statistical significance level was set at 0.05 (5%).

## Results

This review included 38 RCTs, with a total of 18,316 patients. Twenty-five of the 38 trials (65.8%) included over 100 patients, nine of the 38 studies included over 500 participants (23.7%) and one study, over 2000 participants (2.6%). Overall, 69 outcomes were tested using 62 different outcome measures (Supplementary Table 1 and 2 respectively – S.2 and S.3). All but five of the studies (86.8%) assessed improvement in symptoms of OAB: the symptoms assessed were stated clearly in 29 of the 33 (87.9%) whilst the remaining 4 studies (12.1%) assessed efficacy as ‘change in micturition daily variables’, ‘improvement in symptoms’, ‘pre and post-treatment bladder daily variables’ and ‘change in OAB symptoms from baseline’. Some of the OAB symptoms assessed were as follows: change from baseline to week 12 in the number of micturitions per 24 h, the number of nocturia episodes per 24 h and the number of urgency episodes per 24 h. Other symptom related variables included change in volume of the first nocturnal void, level of urgency and the number of patients achieving continence.

Of the 38 RCTs analysed in this review, 25 studies clearly stated the primary outcome in the methods section (65.8%), whilst 23 studies declared the secondary outcomes (60.5%). Of studies that listed primary outcomes, five (13.2%) included more than one outcome. Three studies stated primary but not secondary outcomes (7.9%).

Quality of life was also assessed in over half of the studies (23 studies [60.5%]), either as a primary or a secondary outcome. Examples of QoL assessments included patient satisfaction, improvement in work productivity, change in quantity and quality of sleep and patient perspective on impact of the disease. However, in most trials the outcomes were outlined as changes from baseline to end-of-treatment in symptom-bother scores. Validated questionnaires were used in 29 studies (78.4%); the most commonly used were OAB-q and PPBC (assessed in 10 studies [26.3%]). Other questionnaires used included KHQ, the Patient Perception of Intensity of Urgency Scale (PPIUS), International Prostate Symptom Score (IPSS), Work Productivity and Activity Impairment questionnaire (WPAI), Nocturia Quality of Life questionnaire (N-QoL), Incontinence Quality of Life questionnaire (I-QoL) and others. QoL related outcomes were identified as primary outcomes in 2 studies (5.3%).

Safety was assessed in 28 studies (73.7%) and was stated as a primary outcome in one study; this was primarily assessed as incidence and severity of treatment emergent adverse effects (TEAEs). Other safety parameters were also assessed including side effects of antimuscarinics such as dry mouth and constipation, hypersensitivity reactions, hyponatraemia, cardiac effects, pruritus, nausea and vomiting, headaches, cognitive effects and death among others. Discontinuation rates were also documented in 13 studies (34.2%). Safety of the drugs used was monitored objectively and subjectively, through patients reporting side effects at clinic appointments, as well as through laboratory tests, ECGs, physical examinations and urinalyses among others.

Other outcomes assessed include changes to maximum flow rate, post-void residual volume, post-void dribbling, voiding efficiency, change from baseline to week 12 in transvaginal ultrasound bladder wall thickness and difference in the Hopkins Verbal Learning Test – Revised (HVLT-R) scores from baseline to week 4.

Twenty-five of the studies (65.8%) received commercial funding. The remaining 13 studies that did not receive commercial funding (34.2%) enrolled 1111 women (6.06%) receiving drugs or placebo therapy, and none of these included more than 148 participants. Twelve studies (31.6%) were published in general journals such as ‘Contemporary Clinical Trials’; four (10.5%) were published in specialist journals such as ‘Menopause’ and the remainder (57.9%) were published in subspecialist journals such as ‘International Journal of Urology’ and ‘European Urology’.

No statistically significant difference was found between journal type and JADAD (*P* = 0.102) or MOMENT scores (*P* = 0.224) shown in Table 1. The most commonly seen JADAD score for general (*n* = 7; 58.3%) and specialist journals (*n* = 2; 66.7%) was 3, whilst for sub-specialist journals the score was 5 (*n* = 12; 52.2%). The most frequently reported MOMENT score was 6, although most general journals received a score of 3 (*n* = 5; 41.7%).

**Table 1:** The correlation between MOMENT, JADAD, journal type, commercial funding, year of publication and impact factor. The statistical significance is set to 0.05 (5%)

	Commercial funding	Journal type	Year	IF	JADAD
JADAD	0.066	0.102	0.298	0.263	
MOMENT	0.111	0.224	0.224	0.536	0.000

Furthermore, there was no statistically significant correlation between commercial funding and JADAD or MOMENT scores (*P* = 0.066 and *P* = 0.111, respectively).

In addition, no correlation was found between JADAD or MOMENT scores, IF or year of publication (Table 1). However, there does appear to be a statistically significant correlation between MOMENT (outcome reporting quality) and JADAD (methodological quality) scores at the level of 0.05 (Spearman’s rho = 0.548).

## Discussion

### Main findings

This SR evaluated outcome reporting in RCTs on the pharmacological therapies for OAB. Tolterodine and solifenacin were the most commonly used pharmaceutical agents in many of the trials. Placebo was used as a control in 19 studies (50.0%). Seven studies evaluated more than two interventions (18.4%).

A wide range of outcomes were tested, mainly efficacy (86.5%) assessed through bladder diaries followed by QoL. However, work productivity, patient satisfaction, cognitive function and retreatment probability were reported only once (2.7%). This could be due to the presence of standardized forms that are completed by patients and easily analysed, thus representing reliable tools from which data can be collected. This differs from outcomes such as ‘comfortability with continuing medication’ which would be rather difficult to quantify and subsequently more difficult to analyse. Emphasis has to be put on the fact that cognitive function was only analysed in one RCT.

Sexual function was underrepresented having only been assessed in one study (2.7%). This was surprising as OAB is known to affect sexual health of women due to the need to wear incontinence pads or having urinary symptoms during intercourse, thereby resulting in low libido and diminished sexual activity [25]. There appears to be a lack of RCTs carried out on the effect of pharmacological therapy on female sexual dysfunction.

Although correlation between JADAD/MOMENT scores and journal types were not statistically significant, trials published in sub-specialist journals generally received higher scores. Caution is needed when interpreting data regarding the specialist journals, as only three were included within the review, thus each study represents 33.3%. Additionally, a statistically significant correlation between MOMENT and JADAD scores indicates that overall methodological quality of the study is usually associated with outcome reporting quality.

A number of trials did not clearly identify both primary and secondary outcomes; this can increase the risk of reporting bias and possibly positive outcome bias, due to statistical analysis of multiple outcomes [26]. Furthermore, studies may have been powered for some outcomes but underpowered for some other. Therefore, care must be taken when interpreting results from such studies.

### Strengths and limitations

To our knowledge this is the first SR to create an inventory of outcomes and outcome measures used in OAB trials, particularly relating to pharmacological therapy in women. We applied robust and standardized methodology in line with our previous systematic reviews in multiple areas of pelvic floor disorders (8-21)]. This approach may reduce risks of bias in selection and interpretation of data, and has been used in previous studies with a similar design by several working groups in the field of development of COS [27]. This review contains only RCTs, which are the highest-ranking primary studies due to high internal validity and ability to determine a cause-effect relationship by controlling intervention groups.

A potential limitation of this study stems from the exclusion of observational studies which may report important outcomes with regard to pharmacological therapy. Furthermore, the use of RCTs is associated with certain limitations; patients may have had a strong preference for a specific intervention and may withdraw from a study or comply poorly if not allocated to it (resentful demoralization) [28]. This can result in skewed reporting of adverse events and affect patient perception of the effect of the treatment (performance bias) [28]. In addition, the randomisation technique does not reflect allocation of treatment in a clinical setting, thus potentially threatening the validity of the trial. In order to test the extent to which patient preference can affect outcome, it may be worth carrying out ‘partially randomised patient preference trials’ to compare treatment benefit against those in RCTs. However these limitations are weaknesses inherent to methodology of primary studies rather than to our systematic review.

We also included studies in the English language only – although we may have excluded valuable studies from other geographic areas in the world, we believe it was necessary to do so, so as to avoid taxonomy and terminology issues that could occur with translation. The articles included in the study covered over ten countries, with research from Europe, America and Asia; we therefore believe that primary trials represent wider geographical areas and research priorities.

### Interpretation

The results from this study, showing variations in outcomes and outcome reporting are in agreement with reporting variations identified in previously published SRs in urogynaecological conditions such as stress urinary incontinence and pelvic organ prolapse (POP) among others conducted and published by CHORUS [7–20].

Systematic reviews in other areas of obstetrics and gynaecology have also identified variations in outcome reporting. Hirsch et al. found that outcome reporting in endometriosis varied significantly between trials, making comparison and combination of data into meta-analyses difficult and reduces the extent to which patient care can be enhanced as a result of guideline implementation [29]. This also appears to be the case in other obstetric and gynaecological areas such as fetal growth restriction and hyperemesis gravidarum, highlighting the heterogeneity of outcome reporting in such trials. It is important to remember that outcomes addressed in trials may have different importance to clinicians, patients and policy makers. It is recommended that RCTs encompass all such outcomes in order to produce more meaningful research evidence.

Outcome variation can be explained as a result of intervention variation in the therapy of OAB. Pharmacological agents may be selected according to local hospital guidelines, clinician and patient preferences (including route of administration such as transdermal vs oral), pharmaceutical company influence and patient co-morbidities or contraindications to certain medications. As a result, certain outcomes may be favoured by certain researchers, leading to a variety of outcomes assessed. Choices of outcomes to collect and report may be influenced by biases of individual researchers or different clinical specialty. For instance, gynaecologists may prioritize assessment of lower genital tract changes, whilst urologists may instead prioritize urinary tract related parameters such as micturition diary variables or urodynamic results.

Reporting bias also plays a role in the underrepresentation of adverse events.

### Recommendations

There is currently no consensus regarding which outcomes or outcome measures should be used when carrying out research on OAB. We recommend the implementation of the most frequently reported outcomes and outcome measures from this study as interim core outcome sets while the process of development of core outcome sets is in progress. This is a minimum set of outcomes that could be selected, collected and reported. In addition researchers could collect and report outcomes of their choice in line with their research priorities. Examples of efficacy variables include change from baseline to end-of-treatment in number of urgency incontinence episodes, number of nocturia episodes and micturition episodes. Percentage of patients reaching continence may also be added.

Safety should be monitored as the incidence and severity of TEAEs. Outcomes on cognitive function are clearly underreported and there is limited evidence on the link between anticholinergics and the risk of dementia. QoL can be assessed as changes in validated questionnaire scores. Validated questionnaires that encompass questions relating to both symptoms and QoL may enable better comparisons between outcome measure scores.

We recommend the above outcomes and outcome measures as they are currently the most commonly used in existing research, thus any further research using the same criteria can be synthesized in meta-analyses and SRs to produce valid and reliable results. This recommendation is supported by the International Consortium for Health Outcomes Measurement (ICHOM) Standard Set for Overactive Bladder, which developed recommendations based on outcomes that were of most value to patients, doctors and measurement experts [30].

The development of a core outcome set to overcome current inconsistencies in research will enable the research community to proceed to more robust synthesis of existing and future studies and to ultimately improve clinical practice standards. This SR is the first stage in a process of developing COS and COMS in female idiopathic OAB. Our working group is constructing a staged protocol for the process of development of COS and COMs with the involvement of multiple stakeholders including clinicians from relevant specialities and wider geographical backgrounds, patient representatives, allied healthcare professionals, industry representatives, regulators and professional societies and organizations. This process will include a series of Delphi surveys and consensus meetings as described and recommended by the COMET handbook (reference).

## Conclusion

Our study showed that the most commonly reported outcome domains were efficacy, safety and QoL. The most frequently used outcome measure was micturition diary and the most commonly used validated questionnaires were OAB-q, PPBC and IIQ-7. Therefore, we recommend that these outcomes and measures are implemented in future research.

### Contribution to authorship

SKD conceived the idea, developed the project and supervised all stages of project development and completion including facilitating consensus where required, RM, MR and CD performed data collection, tabulated data and analysis and quality assessments, PS performed statistical analysis, RM drafted the manuscript, all authors reviewed the manuscript, proceeded to consensus and performed revisions of the manuscript and approved the final version.

## Supplementary Information

ESM 1 (DOCX 166 kb)

ESM 2 (DOCX 22 kb)

ESM 3 (DOCX 22 kb)
