# Supplementary material for: Outcome reporting in randomized controlled trials (RCTs) on the pharmacological management of idiopathic overactive bladder (OAB) in women; a systematic review for the development of core outcome sets (COS)
Source: Int Urogynecol J. 2022 Jan 10;33(5):1243–50. doi: 10.1007/s00192-021-05040-1 (PMC9120103; doi:10.1007/s00192-021-05040-1)
Supplement: Supplementary file 1 — (DOCX 166 kb) [file 192_2021_5040_MOESM1_ESM.docx]

# Supporting information

## S.1 PRISMA flow diagram


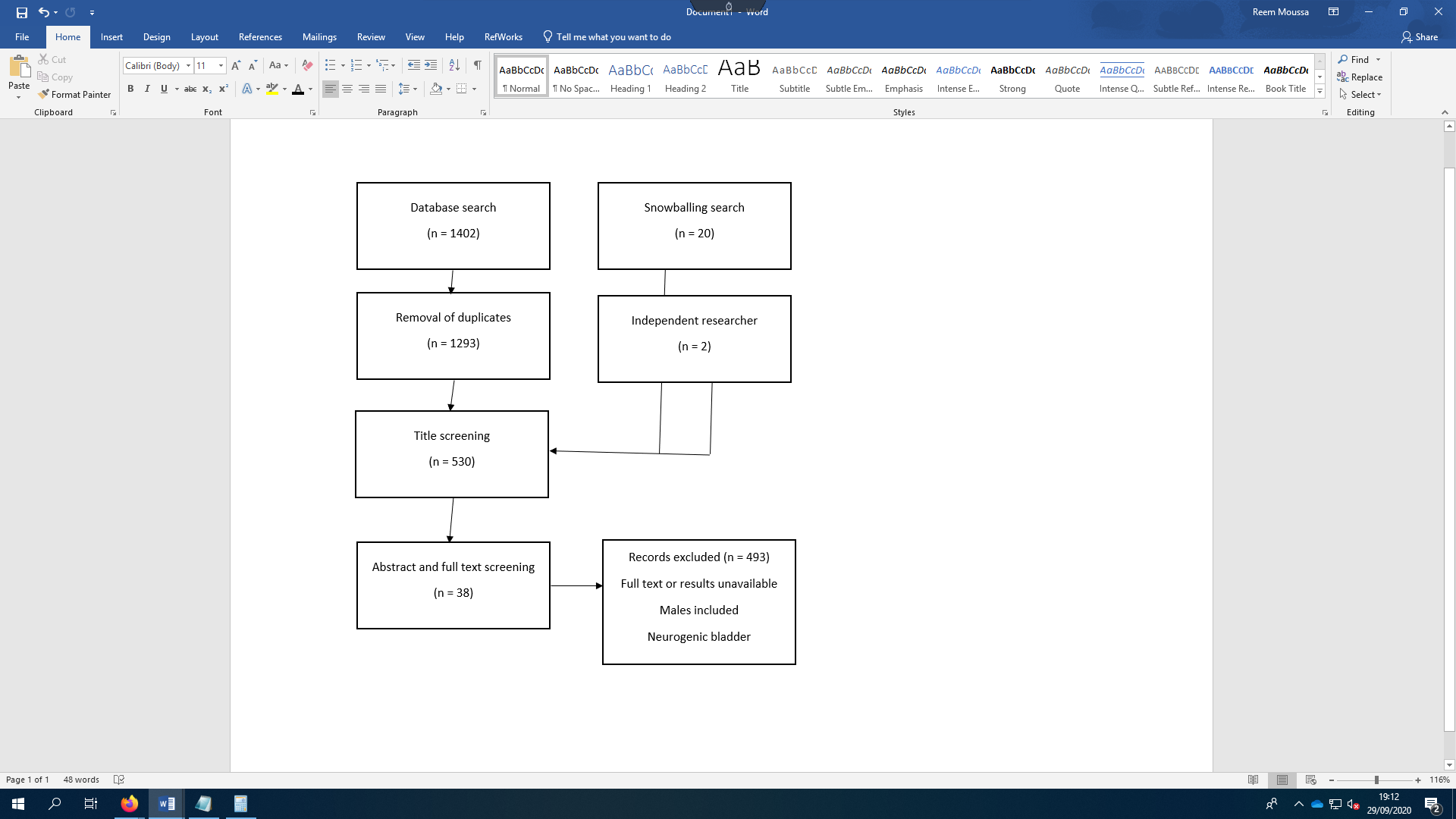


*Figure 1: PRISMA flow diagram showing the process of data collection and narrowing of the search.*

*Trials were excluded if they did not meet the inclusion criteria of idiopathic OAB, pharmacological management and female participants.*
