# Supplementary material for: Outcome reporting in randomized controlled trials (RCTs) on the pharmacological management of idiopathic overactive bladder (OAB) in women; a systematic review for the development of core outcome sets (COS)
Source: Int Urogynecol J. 2022 Jan 10;33(5):1243–50. doi: 10.1007/s00192-021-05040-1 (PMC9120103; doi:10.1007/s00192-021-05040-1)
Supplement: Supplementary file 2 — (DOCX 22 kb) [file 192_2021_5040_MOESM2_ESM.docx]

**S.2 Outcomes, themes and domains**

*Table 1 showing outcomes grouped into themes and domains*

| Theme | Outcome domain | Outcome | Frequency of outcomes reported in each domain |
| --- | --- | --- | --- |
| Clinical effectiveness | Achieving continence |  | 1 |
|  | Improvement in OAB symptoms | Nocturia episodes | 17 |
|  |  | Time for first nocturnal void | 1 |
|  |  | Mean nocturnal void volume | 1 |
|  |  | Mean voiding interval | 1 |
|  |  | First desire to void | 2 |
|  |  | [Daytime] frequency | 13 |
|  |  | Diurnal frequency | 1 |
|  |  | Micturition episodes | 15 |
|  |  | Mean voided volume | 11 |
|  |  | Total voided volume | 6 |
|  |  | Mean number of daily voids | 2 |
|  |  | Mean volume of first nocturnal void | 1 |
|  |  | Urgency incontinence episodes | 20 |
|  |  | Urgency urinary incontinence episodes | 5 |
|  |  | Urgency episodes | 16 |
|  |  | Urgency severity | 2 |
|  |  | Diary-dry rate | 1 |
|  |  | Number of continence pads used | 1 |
|  |  | Post void dribble | 1 |
|  |  | Not specified | 4 |
|  |  | Effect of combined therapy | 1 |
|  | Cost effectiveness |  | 1 |
|  | Responder status |  | 1 |
|  | Vaginal parameters | pH | 1 |
|  |  | Maturation index | 1 |
|  |  | Discharge | 1 |
|  |  | With or without hypertension | 1 |
|  |  | Temperature and improvement in symptoms | 1 |
|  |  | Effect of age on efficacy | 1 |
|  |  | Patient selected goal achievement | 1 |
|  | Clinical testing/urodynamic studies | Post void residual | 3 |
|  |  | Haematology | 1 |
|  |  | Maximal flow rate/detrusor pressure | 2 |
|  |  | Urodynamic variables | 1 |
|  |  | Change in HVLT-R* score | 1 |
|  |  | Overactivity index | 1 |
|  |  | Mean cystometric capacity | 1 |
|  |  | Mean VFOC** | 1 |
|  |  | Mean PFOC*** | 1 |
|  |  | Detrusor pressure on rising | 1 |
|  |  | Conversion to detrusor instability | 1 |
|  |  | Unstable contraction height and pressure rise during cystometry | 1 |
|  |  | Perception threshold of urethra and bladder | 1 |
|  |  | Current perception threshold value | 1 |
|  | Refusal of urodynamic studies after treatment |  | 1 |
|  | Probability of retreatment |  | 1 |
|  | Urinary nerve growth factor |  | 2 |
|  | uBDNF^+^ |  | 1 |
| Quality of life of patient | Not specified |  | 9 |
|  | Sleep |  | 5 |
|  | Work productivity |  | 1 |
|  | Patient reported outcomes | Validated questionnaire scores | 14 |
|  | Patient satisfaction with treatment |  | 1 |
|  | Patient perspective of disease |  | 1 |
| Adverse events | Pharmacological adverse events | Antimuscarinic-related adverse events | 29 |
|  |  | Headaches | 4 |
|  |  | Cognitive function | 1 |
|  |  | Prolonged QT interval | 1 |
|  |  | Hepatotoxicity | 1 |
|  |  | Nephrotoxicity | 1 |
|  |  | Hypertensive event | 1 |
|  |  | Back pain | 1 |
|  |  | Sinusitis | 1 |
|  |  | Arthralgia | 1 |
|  |  | Dyspepsia | 1 |
|  |  | Abdominal pain | 1 |
|  |  | Death | 2 |
|  |  | Severity of TEAEs ^++^ | 2 |
|  | Discontinuation rate |  | 16 |
|  | Tolerability | Comfortability with continuing medication | 3 |
|  |  | Dose changes | 1 |
|  |  | Compliance | 2 |
|  |  | Not specified | 2 |

*Key: HVLT-R* (Hopkins verbal learning test – revised) VFOC** (volume in first overactive contraction) PFOC*** (pressure in first overactive contraction) uBDNF^+^ (urinary brain-derived neurotrophic factor) TEAEs^++^ (treatment emergent adverse events)*
