# Supplementary material for: Outcome reporting in randomized controlled trials (RCTs) on the pharmacological management of idiopathic overactive bladder (OAB) in women; a systematic review for the development of core outcome sets (COS)
Source: Int Urogynecol J. 2022 Jan 10;33(5):1243–50. doi: 10.1007/s00192-021-05040-1 (PMC9120103; doi:10.1007/s00192-021-05040-1)
Supplement: Supplementary file 3 — (DOCX 22 kb) [file 192_2021_5040_MOESM3_ESM.docx]

**S.3 Outcome measures**

*Table 2: table of reported outcome measures*

| **Outcome** | **Outcome measure** | | **Number of studies** |
| --- | --- | --- | --- |
| Safety | Physical examination and paraclinical investigations | Examination | 4 |
|  |  | Ethnicity | 2 |
|  |  | Height and weight | 1 |
|  |  | BMI | 2 |
|  |  | Age | 2 |
|  |  | Vital signs | 6 |
|  |  | ECG | 5 |
|  |  | Urinalysis | 3 |
|  | Reporting of TEAEs | Reporting in clinic/over the phone | 4 |
|  | Laboratory testing | Electrolytes | 1 |
|  |  | Renal function | 2 |
|  |  | Liver function | 1 |
|  |  | Pregnancy test | 2 |
|  |  | Plasma prolactin | 1 |
|  |  | Not specified | 2 |
| Efficacy | Micturition diary | 1 day | 1 |
|  |  | 2 day | 1 |
|  |  | 3 day | 22 |
|  |  | 3 day eDiary | 1 |
|  |  | 5 day | 1 |
|  |  | 7 day | 1 |
|  | Validated questionnaires | Overactive Bladder Symptom Score | 9 |
|  |  | Hopkins Verbal Learning Test-Revised | 1 |
|  |  | Mini Mental State Exam | 1 |
|  |  | Mini Mental Status X | 1 |
|  |  | Trail Making Test A & B | 1 |
|  |  | Digit span | 1 |
|  |  | ICIQ-M/FLUTSsex | 1 |
|  | Clinical testing | Urine culture | 1 |
|  |  | Urodynamic studies | 9 |
|  |  | Neurometer constant current stimulator | 1 |
|  |  | Urinary nerve growth factor | 1 |
|  | Ultrasound | Transvaginal | 2 |
|  |  | Transabdominal | 3 |
|  |  | Bladder wall thickness | 1 |
|  | Performance based outcome | Twenty minute pad test | 2 |
|  | Vaginal indices |  | 1 |
|  | Post Void Residual volume | Ultrasound | 1 |
|  |  | Not specified | 3 |
|  | Urodynamics | Detrusor pressure | 1 |
|  | Stimulated salivary flow |  | 1 |
| Quality of life | Validated questionnaires | Patient Perception of Bladder Control | 10 |
|  |  | Overactive bladder questionnaire | 10 |
|  |  | Incontinence Impact Questionnaire-7 | 7 |
|  |  | Health Related Quality of Life | 7 |
|  |  | Urinary Distress Inventory-6 | 6 |
|  |  | King’s Health Questionnaire | 5 |
|  |  | Epworth Sleepiness Scale | 3 |
|  |  | Pittsburgh Sleep Quality Index | 3 |
|  |  | International Prostate Symptom Score | 2 |
|  |  | Patient Global Impression of Improvement-Incontinence | 2 |
|  |  | Urgency Perception Score | 2 |
|  |  | Incontinence-Quality of Life | 1 |
|  |  | Medical Care and Use Index | 1 |
|  |  | Nocturia-Quality of Life | 1 |
|  |  | Indevus Urgency Severity Scale | 1 |
|  |  | Patient Perception of Intensity of Urgency Scale | 1 |
|  |  | Quality of Life-questionnaire | 1 |
|  |  | EQ-5D-5L | 1 |
|  |  | Nocturia Impact-diary | 1 |
|  |  | Urge Urogenital Distress Inventory | 1 |
|  |  | Urge Incontinence Impact Questionnaire | 1 |
|  |  | Work Productivity and Activity Impact questionnaire | 1 |
|  |  | Benefit Satisfaction and Willingness to continue measure | 1 |
|  |  | Pelvic Organ Prolapse-Distress Inventory | 1 |
|  | Visual Analogue Scale (VAS) | Not specified | 2 |
|  |  | VAS 0-100 | 3 |
|  |  | VAS 0-10 | 1 |
|  |  | Treatment Satisfaction-VAS | 2 |
|  |  | Urgency Bother-VAS | 1 |
|  | Patient reported questionnaire |  | 5 |
| Miscellaneous | Responder analyses | Full analysis set | 2 |

*Key: ICIQ/FLUTSsex (International Consultation on Incontinence Questionnaire – Female Lower Urinary Tract Symptoms sex) EQ-5D-5L (EuroQol-5D 5-level)*
